# A Narrative Overview of Current Anesthetic Drugs in Electroconvulsive Therapy

**DOI:** 10.3390/life11090981

**Published:** 2021-09-18

**Authors:** Kevin Lee, Kimberly D. Jenkins, Tanaya Sparkle

**Affiliations:** Department of Anesthesiology, University of Toledo Medical Center, Toledo, OH 43614, USA; Kevin.Lee2@UToledo.Edu (K.L.); kimberly.jenkins2@utoledo.edu (K.D.J.)

**Keywords:** anesthesia, ECT, ketamine, depression, electroconvulsive therapy

## Abstract

Electroconvulsive therapy (ECT) is a definitive treatment for patients with psychiatric disorders that are severe, acute, or refractory to pharmacologic therapy. Providing anesthesia for ECT is challenging, as the effect of drugs on hemodynamics, seizure duration, comfort, and recovery must be considered. We highlight and aim to review the common anesthetics used in ECT and related evidence. While drugs such as methohexital, succinylcholine, and etomidate have been used in the past, other drugs such as dexmedetomidine, ketamine, and remifentanil may provide a more balanced anesthetic with a greater safety profile in select populations. Overall, it is essential to consider the patient’s co-morbidities and associated risks when deciding on an anesthetic drug.

## 1. Introduction

With over 100,000 electroconvulsive therapy (ECT) treatments performed annually in the United States, ECT has been a mainstay treatment option in patients with severe or acute psychiatric disorders refractory to pharmacological therapy [1]. Greater application of the current practices in anesthetic management and perioperative care for ECT will improve patient outcomes and reduce variability in clinical practice. This narrative review summarizes the currently used pharmacologic agents used to provide anesthesia safely and effectively for ECT. Thereby, the importance lies within investigating the potential impact on patient mortality, improvement in perioperative clinical practice, and future translational studies.

The use of anesthetic drugs for ECT requires a delicate balance between providing adequate anesthesia and minimal drug-related post-operative side effects while maintaining stable hemodynamic parameters and having a limited effect on seizure duration. The use of muscle relaxants has been a mainstay to facilitate ECT treatments. The role of muscle relaxants in ECT stems from the ability to reduce fractures. It has been documented in the literature that during ECT, 35% of patients who do not receive muscle relaxants sustain fractured vertebrae. The implementation of short-term paralytics, such as succinylcholine, mitigates this while also minimizing residual paralysis in the post-anesthesia care unit. Luccarelli, Henry, and McCoy noted a fracture rate of 3.56 per 1 million treatments in their study when muscle relaxants were used [2].

Electroconvulsive therapy (ECT) has been a well-established treatment option for patients who suffer from severe and medication-resistant depression, mania, schizoaffective disorder, catatonic symptoms, and acute suicidal ideations [3]. The underlying mechanism for which ECT implores its therapeutic effect is by provoking a generalized epileptic seizure. More specifically, the goal is to induce a motor and electroencephalogram (EEG) seizure lasting at least 25 and 40 s, respectively [4]. Treatments are usually performed three times a week for a total of six to twelve treatments, and maintenance therapy can be performed once a week to once a month [3]. Anesthesia is provided during ECT for sedation and muscle relaxation. However, the choice of anesthetic agents for sedation is critical, as it can affect the seizure quality, seizure duration, and post-procedure course [5,6]. Transcutaneous electrodes are applied either unilaterally or bilaterally, and 70 to 120 volts of pulsed electricity are used to induce a seizure, ideally lasting 30 s [7]. Anesthesia is provided during ECT for sedation and muscle relaxation. 

Furthermore, it is essential to understand the physiologic response that occurs when ECT is performed. When an electrical impulse is applied via transcutaneous electrodes to induce a seizure, an acute cardiovascular response occurs. This cardiovascular response is initially parasympathetic and associated with bradycardia lasting 10 to 15 s [8,9]. Subsequently, a sympathetic response occurs in which tachycardia and hypertension occur via a surge in catecholamine release, lasting as long as five minutes. It has been documented that blood pressure can be increased by 30% to 40%, and heart rate can be increased by 20% [8,9]. This attenuated sympathetic response has apparent sequelae, as this can lead to arrhythmias, myocardial ischemia, cerebral hemorrhage, and cortical blindness.

Through this narrative review, we hope that application of newer drugs based on evidence can improve patient morbidity and perioperative safety.

## 2. Methodology

We performed a literature search in the PubMed and Ovid MEDLINE databases, with the last search done on 30 June 2021 to compile current literature for our narrative review. A search using keywords ‘Electroconvulsive Therapy’ returned 15,709 articles in the PubMed Database, which was narrowed down to 1153 results with the keyword Anesthesia. Results from 2010 (476) were reviewed to focus on publications relevant to newer drugs and practices related to Anesthesia for Electroconvulsive therapy. Articles related to adult patients who underwent ECT under anesthesia provided by an anesthesia-trained physician were included. Articles that systematically analyzed smaller studies were also included, along with any studies that the authors felt valuable to providing a broad perspective on the topic. Although we acknowledge the risk of introducing significant selection bias, being a narrative overview, we intend to summarize the latest information related to various drugs used by the Anesthesiology team and understand its implications. Information related to the agent’s effect on seizure duration, hemodynamic parameters, postoperative recovery, and pharmacology were summarized for inclusion in the article.

## 3. Anesthetic Drugs Currently Used during ECT

An ideal anesthetic used for sedation during ECT must have some specific characteristics. It must have a quick onset, easy administrative capability, short distribution half-life, and minimal effect on memory, seizure duration, and postoperative recovery [10]. Table 1 summarizes the drugs commonly used by the anesthesia team, as well as their pharmacology and post-procedural considerations while in recovery.

### 3.1. Etomidate

Etomidate mediates its anesthetic effects in the central nervous system by acting as a γ-aminobutyric acid (GABA) type A agonist. An intravenous bolus dose of 0.2 mg/kg to 0.4 mg/kg is typically sufficient to achieve a hypnotic effect. Metabolism of etomidate is primarily via hepatic esterase activity, which hydrolyzes the drug into a carboxylic acid and is subsequently excreted in the urine. As a result, elderly or ill patients may require lower dosing because of reduced protein binding and reduced clearance. The total plasma clearance is 15–20 mL/kg/min, and the terminal metabolic half-life of etomidate in humans ranges from 2–5 h [11]. These specific properties of etomidate are summarized in Table 1.

Etomidate is also commonly used in ECT as it increases seizure duration. However, it is adversely associated with myoclonus, a longer time to wake up when compared to methohexital, and limited blunting from hypertension or tachycardia [8,9].

A systematic review and meta-analysis by Singh et al. compared etomidate’s effect on seizure duration with propofol, thiopental, and methohexital [12]. Seventeen trials were identified involving 704, 84, 2491, and 258 settings of ECT using etomidate, methohexital, thiopental, and propofol, respectively. In the etomidate group, pooled EEG seizure duration was longer by 2.23 s (95% confidence interval [CI], −3.62 to 8.01; *p* = 0.456) than methohexital, longer by 17.65 s (95% CI, 9.72–25.57; *p* < 0.001) than propofol, and longer by 11.81 s (95% CI, 4.26–19.35; *p* = 0.003) than thiopental. Pooled motor seizure duration was longer in the Etomidate group by 1.45 s (95% CI, −4.79 to 7.69; *p* = 0.649) than methohexital, longer by 11.13 s (95% CI, 6.64–15.62; *p* < 0.001) than propofol, and longer by 3.60 s (95% CI, 2.15–5.06; *p* < 0.001) than thiopental. Therefore, when comparing EEG and motor seizure durations between etomidate with propofol and thiopental, etomidate is more advantageous in the setting of ECT. Etomidate, however, displayed equivocal results in comparison to methohexital seen in this study.

### 3.2. Methohexital

Similar to etomidate, methohexital acts on the GABA_A_ receptor of the central nervous system. The induction dose of methohexital to achieve sufficient anesthesia for ECT is 1.5 mg/kg. Metabolism of methohexital occurs in the liver through demethylation and oxidation, and the metabolites are excreted through the kidneys. Because methohexital has a quick onset and a fairly short half-life of 5.6 ± 2.7 min, it is an ideal anesthetic for short procedures such as ECT [13,14]. These specific properties of methohexital are highlighted in Table 1.

Methohexital is considered by many to be the gold standard for ECT. It does not affect seizure duration while also blunting the sympathetic hemodynamic response commonly seen in ECT [8,9].

### 3.3. Dexmedetomidine

Dexmedetomidine is a highly selective α2-adrenergic receptor agonist and has been documented to be 8 to 10 times more selective than clonidine towards the α2 receptor. As discussed, dexmedetomidine is a valuable sedative agent with analgesic properties, favorable hemodynamics, and minimal respiratory depression. Dexmedetomidine undergoes almost complete biotransformation through direct N-glucuronidation and cytochrome P-450 (CYP 2A6)-mediated aliphatic hydroxylation to inactive metabolites and is excreted almost entirely in urine. A premedication dose of 0.33 to 0.67 mg/kg IV can be given 15 min before surgery, but the provider should be aware of bradycardia and hypotension when bolus doses are given. The recommended dose range of 0.2 to 0.7 μg/kg/h was administered as an intravenous infusion. Finally, the distribution phase is rapid, with a half-life of distribution of approximately 6 min and an elimination half-life of 2 h [15]. An overview of these specific properties of dexmedetomidine can be seen in Table 1.

In a prospective, randomized, double-blinded crossover study by Sannakki et al., it was found that intravenous infusion of 1 µg/kg of dexmedetomidine before induction of anesthesia was effective in preventing the acute hyperdynamic response without altering seizure duration [16]. Specifically, it was found that in the dexmedetomidine group that heart rates at the 3rd and 5th min after electric stimulus were 4.5 ± 20.1 and 90.4 ± 12.8/min as compared to 111.9 ± 15.5 and 109.0 ± 13.7 in Group N, respectively (*p* < 0.0001). Regarding systolic blood pressure, the dexmedetomidine group had better systolic control with a 116.53 ± 26.09 compared to the control group of 138.03 ± 19.32 (*p* < 0.001). However, patients may have delayed recovery and delayed discharge based on prolonged Modified Aldrete’s Score and Richmond Agitation-Sedation Score seen in the dexmedetomidine group. In a randomized, double-blind study by Bagle et al., Dexmedetomidine was found to better attenuate the hyperdynamic response commonly seen in ECT with minimal effect on seizure duration or recovery time [17]. A systematic review and metanalysis was performed by Li et al. to identify the effects of using dexmedetomidine in combination with intravenous anesthetics, for ECT, on seizure duration, hemodynamic parameters such as maximum mean arterial pressure (MAP) and heart rate after ECT, recovery time, and post-ECT agitation. Six studies with 166 patients undergoing 7706 ECT sessions were analyzed. They concluded that there was no significant difference in seizure duration between the dexmedetomidine and non-dexmedetomidine groups (motor seizure duration: 6 studies; *p* = 0.41; EEG seizure duration: 3 studies; *p* = 0.58). They identified that the maximum MAP and heart rate after ECT were significantly lower in the dexmedetomidine group (MAP: 6 studies; *p* = 0.009; H.R.: 6 studies; *p* = 0.001). They also found that adding dexmedetomidine to the medications used did not significantly prolong recovery time when the reduced-dose propofol was used (4 studies, *p* = 0.12) [18].

### 3.4. Ketamine

Ketamine is a N-Methyl-D-Aspartate (NMDA) receptor agonist. Ketamine is metabolized through N-demethylation to the active form, norketamine. It is this metabolite that allows ketamine to have hypnotic, analgesic, dissociative, and anti-depressive effects. Because ketamine is very lipid-soluble and has a relatively low protein binding, a very large volume of distribution is attained and can rapidly cross the blood–brain barrier. Intravenous administration provides anesthesia within 1–2 min and lasts approximately 20–60 min. Unlike some of the pharmacologic agents, ketamine can be administered intramuscularly, which is beneficial in combative patients. Through the intramuscular route, anesthesia develops within 10–15 min and typically lasts for 30–120 min. In adults, a typical intramuscular dose is 6.5 to 13 mg/kg in adults, whereas a typical intravenous induction dose is 1 to 2 mg/kg [18,19]. These properties of ketamine are summarized in Table 1.

A systematic review and meta-analysis done by Ren et al. found that ketamine in ECT showed no better depression response and remission rates with increased cardiovascular and psychiatric adverse events. Their study did find that although ketamine used in ECT cannot reduce the depressive symptoms at the end of treatment, it could accelerate the anti-depressive effect in depressive patients receiving ECT, especially in those who used ketamine as an add-on anesthetic [20]. Another metanalysis by Ainsworth et al. included 18 double-blinded randomized controlled trials comparing ketamine to other anesthetic medications. They found that ketamine did not improve depressive symptoms early during the course of ECT or later on after the treatment series. Ketamine increased seizure duration (*p* = 0.038) both by itself and when given with other anesthetic medications, even when the electrical dose was decreased. Ketamine monotherapy showed an increase in hypertension compared to other groups, but did not increase any other adverse effects. Although the meta-analysis did not reveal any significant neurocognitive benefit, the authors mentioned that this was limited by the sample sizes and heterogeneity of the sample populations [21]. An older meta-analysis of 17 Randomized Controlled Trials reported similar results, stating that ketamine alone did not appear to improve the efficacy of ECT but, in combination with other anesthetics, may confer a short-term improvement in depressive symptoms [22].

Guerrier et al. did a systematic review and meta-analysis (7 studies out of 38 eligible studies) looking at the effect of Bispectral Index monitor (BIS) numbers on different parameters during ECT, including electrical and motor seizure durations. They found that higher values of pre-ictal BIS levels were associated with longer motor (correlation 0.72, 95% CI (0.29–0.91)) and electrical seizure durations (correlation 0.61, 95% CI (0.39–0.75)) [23]. This finding is interesting considering the known effect of ketamine on increasing the BIS value [24]. Further studies are required to illustrate any direct correlation between BIS value and seizure duration while accounting for the dose of ketamine and the effect of ketamine on response entropy (RE) and state entropy (S.E.).

Studies and expert opinions also suggest that the inclusion of dexmedetomidine, ketamine, or a combination in suitable patients as an adjunct anesthetic can attenuate the sympathetic response, improve patient satisfaction, and is safe to use for ECT [25].

## 4. Other Drugs Used for ECT

### 4.1. Opioids

Opioids have been widely evaluated for use in ECT. Most of the opioids are longer-acting and, therefore, are not suitable for use in ECT. Opioids do not generally increase seizure threshold and help control hemodynamic parameters, making them an excellent adjunct to other anesthetic medications. Opioids are mu, delta, and kappa receptor agonists. In the setting of ECT, the primary clinical effect is on mu receptors by providing analgesia, sedation, and decreased smooth muscle tone.

Remifentanil

Remifentanil is an ultra-short-acting opioid with a quick onset of action and a rapid metabolism by non-specific esterases related to hydrolysis. After a three-hour infusion, the context-sensitive half-life of remifentanil is 3.2 ± 0.9 min, and the maximum time to onset after a single dose is 1–2 min [26,27,28]. An intravenous dose of 1 µg/kg as an adjunct may be used to reduce the amount of methohexital and propofol required to induce unconsciousness. A summary of remifentanil’s properties can be seen in Table 1.

A meta-analysis of 13 RCTs (380 patients and 1024 ECT sessions) looked at seizure duration, maximum systolic blood pressure (SBP), and other parameters. They found that the remifentanil group showed a significantly prolonged seizure duration (motor seizure duration: nine studies, *p* = 0.02; electroencephalogram seizure duration: eight studies, *p* = 0.02). The maximum SBP was also significantly lower in the remifentanil group (seven studies, *p* = 0.02). They also performed a sub-group analysis due to the heterogeneity of the samples for seizure duration analysis. They reported prolonged seizure duration only when the use of anesthetic dose was reduced in the remifentanil group due to its contributory sedative and analgesic properties [29]. Furthermore, a study by Glavez et al. looking at the effect of adjuvant remifentanil on the quality of seizures indices, such as time to slow-wave activity, amplitude, regularity, stereotypy, post-ictal suppression, and duration, did not reveal any significant effect findings [30].

Alfentanil

In addition to remifentanil, alfentanil has also been studied and used in ECT due to a relatively quick maximum time of onset of 1.4 min, a context-sensitive half-life of 47.3 ± 12 min after a three-hour infusion, and a short elimination half-life (1.5 h) compared to other opioids. In an observer-blinded, prospective, randomized crossover study, it was found that a combination of methohexital/alfentanil had a more significant seizure duration than propofol alone [31]. However, recovery time was greater in the methohexital/alfentanil and methohexital groups when compared to propofol alone [8,31]. A combination of remifentanil and methohexital was also evaluated. It was found that the methohexital-sparing effect of remifentanil prolonged seizure duration when using methohexital alone [8,31]. Interestingly, the hemodynamic and recovery times were similar in both groups [8,31]. On the contrary, fentanyl was found to have a limited role in ECT. Mainly because when used with methohexital, seizure duration was reduced, and fentanyl also failed to attenuate the hemodynamic response [8,32].

Thus, shorter-acting opioids, such as alfentanil and remifentanil, have been seen to reduce the amount of methohexital required and, consequently, serve as an adjunct to prolonging seizure duration.

### 4.2. Depolarizing Neuromuscular Blockers

Muscle relaxation agents are utilized in ECT to prevent musculoskeletal injuries, such as fractures and myalgias from the vigorous activity experienced from the seizure [8]. As mentioned previously, Luccarelli, Henry, and McCoy noted a fracture rate of 3.56 per 1 million treatments with the implementation of paralytic agents [2]. Ideally, a short-term paralytic with rapid onset is used so that minimal residual paralysis is seen after the procedure. Succinylcholine is, therefore, the most commonly used paralytic during ECT. It is a depolarizing neuromuscular blocking agent which depolarizes the motor end plate by adhering to post-synaptic cholinergic receptors, resulting in a phase 1 (depolarizing phase) and an eventual phase 2 (desensitizing phase) block.

Succinylcholine typically has an onset of 0.5 to 1.0 min, lasting 5 to 10 min, and the standard dosing is between 0.5 to 1.5 mg/kg. An additional benefit of using a paralytic agent to prevent airway obstruction is that it will help assist the anesthesia provider in mask ventilation during the procedure. Succinylcholine should, however, be used cautiously in patients with bradyarrhythmias [8]. It is well-established that larger and repetitive doses of succinylcholine can potentiate bradycardia. Additionally, succinylcholine should be avoided in patients with a history of malignant hyperthermia and neuromuscular disease. Succinylcholine can cause a life-threatening hypermetabolic skeletal disease associated with hypercapnia, hyperpyrexia, hyperkalemia, and metabolic acidosis in the above diseases, leading to cardiovascular instability. These specific properties of succinylcholine are summarized in Table 1.

### 4.3. Non-Depolarizing Neuromuscular Blockers

In patients in which a short-term depolarizing paralytic such as Succinylcholine is contraindicated, non-depolarizing neuromuscular blockers can be used instead.

#### 4.3.1. Rocuronium

Rocuronium is a non-depolarizing neuromuscular blocking agent that serves as a competitive antagonist to the acetylcholine receptor, preventing acetylcholine from binding and subsequent motor end plate potentials. The dose to provide adequate paralysis is 0.6 mg/kg, and the onset for paralysis is usually 3–5 min [33,34]. Because rocuronium is a longer paralytic, a couple of considerations should be highlighted. Firstly, with any paralytic agent, establishing mask ventilation is essential once the patient is sedated before giving rocuronium. Secondly, because the half-life of rocuronium is 20–45 min, this medication will typically outlast the duration of the procedure. Thus, neuromuscular monitoring is needed to evaluate the amount of residual paralysis the patient has. Table 1 highlights these specific properties of rocuronium. Patients will benefit from reversal with sugammadex due to its rapid onset of action and low side-effect profile. H. Mirzakhani et al. noted that 2 mg/kg of sugammadex is sufficient to reverse rocuronium at a post-tetanic count of 1 or 2 [35]. Inadequate reversal of rocuronium with neostigmine (a cholinesterase inhibitor) and its dosing limitations can cause residual paralysis, resulting in respiratory complications while in the recovery unit. It should also be noted that more than 50% of anaphylactic and anaphylactoid reactions on induction are attributed to rocuronium and other paralytics in this class (non-depolarizing neuromuscular blocking drugs) [33,34,35]. Additionally, rocuronium is eliminated predominately via the hepatobiliary system and partly via the renal system. Finally, acute burn victims and patients suffering from stroke may be resistant to rocuronium and may have inadequate paralysis, although this might not be relevant to the population receiving ECT [33,34]. A case report by Takazawa et al. compared succinylcholine and rocuronium as neuromuscular blocking agents for electroconvulsive therapy in a patient with pseudocholinesterase deficiency [25]. While it would be expected that paralysis with the administration of succinylcholine in a patient with pseudocholinesterase deficiency would be prolonged, Takazawa et al. concluded that the recovery time from muscle relaxation after succinylcholine administration was remarkably longer than that after rocuronium-sugammadex administration. In their case report, seizure duration was unaffected while the time to shifting from the OR was nearly 10 min longer when using Succinylcholine than after rocuronium-sugammadex (*p* < 0.05). Furthermore, in patients without pseudocholinesterase deficiency, it has been shown that 8 mg/kg sugammadex produced equally rapid recovery from ROC-induced (0.6 mg/kg) muscle relaxation compared with spontaneous recovery from 1 mg/kg SCC during ECT [35,36].

#### 4.3.2. Mivacurium

Mivacurium, when available, is a common alternative for patients undergoing ECT in which Succinylcholine is contraindicated. A dose of 0.16 mg/kg to 0.20 mg/kg has been recommended for sufficient paralysis. However, it tends to have a duration of action of 12–20 min with an onset time of 2–3 min. Mivacurium is metabolized by pseudocholinesterase, and a prolonged duration of action will be seen in patients with pseudocholinesterase deficiency. Finally, it is also commonly associated with clinically significant histamine release and occasional hypotension [8].

## 5. Conclusions

This broad narrative review has summarized the drugs currently used worldwide for ECT. It has identified areas requiring further translational investigation by investigating the multitude of pharmacologic agents and their combination for ECT. Methohexital and succinylcholine combination is most commonly used in the United States due to their short duration of action and minimal effects on seizure duration. We cannot recommend a specific combination of drugs due to the differences in patient factors, availability of drugs, institutional culture, and anesthesiologist and psychiatrist preference. However, based on the positive results of smaller studies, we suspect that with more extensive randomized and prospective studies, the inclusion of ketamine and dexmedetomidine as adjuncts may help safely limit the dose of other anesthetics and eventually phase out prior anesthetic management with ECT.

## Figures and Tables

**Table 1:** Summary of drugs currently used during anesthesia for ECT.

Drug	Onset of Action	Half-Life	Issues during Postprocedural Recovery	Effect on Seizure Duration
Etomidate	30–60 s	2–5 h	Nausea, vomiting, adrenal suppression, clonus, and injection site pain.	Increase
Methohexital	30–60 s	3–6 h	Acute intermittent porphyria, drowsiness, apnea, confusion, nausea, and vomiting.	No effect
Dexmedetomidine	4–5 min	2–2.5 h	Bradycardia, arrhythmias, and hypotension.	No effect
Ketamine	1–2 min	2.5–3 h	Disassociation, increased secretions, hypertension, and tachycardia.	Increase
Remifentanil	1–3 min	1–20 min	Dyspnea, hypotension, bradycardia, nausea, and vomiting.	No effect
Succinylcholine	30–60 s	30–60 s	Apnea, residual paralysis, malignant hyperthermia, and bradycardia.	No effect
Rocuronium	3–5 min	20–45 min	Respiratory complications, if not entirely reversed, and anaphylaxis.	No effect

## Data Availability

No new data were created or analyzed in this study. Data sharing is not applicable to this article.
